# Supplementary material for: Transposon signatures of allopolyploid genome evolution
Source: Nat Commun. 2023 Jun 1;14:3180. doi: 10.1038/s41467-023-38560-z (PMC10235133; doi:10.1038/s41467-023-38560-z)
Supplement: Supplementary file 1 — Supplementary Information [file 41467_2023_38560_MOESM1_ESM.pdf]

Transposon signatures of allopolyploid subgenome evolution

Adam Session and Daniel S. Rokhsar

Supplementary Note 1. Identification of *Brassica napus* subgenome-specific 13-mers

We used Jellyfish[1] to count the 13-mers of the *Brassica napus* chromosomes from assembly Brana_ZS_PB_V1.0[2]. The tetraploid *B. napus* genome (2*n*=4*x*=38) is divided into two subgenomes with *n*=10 for the A subgenome, and *n*=9 for the C subgenome.

We used the synteny analysis of Song *et al*. to identify initial homoeolog pairs[3]. Due to a number of rearrangements between the subgenomes, we used five pairs of homoeologous chromosomes (A1-C1, A2-C2, A4-C4, A5-C5, and A9/A10-C9) to identify 13-mers that are enriched in one or the other subgenome (Note that A9 and A10 together are homoeologous to C9). We computed the 13-mers that were (1) found in at least 100 copies in the entire tetraploid genome, and (2) at least two-fold enriched in one member of each of the five chromosome pair listed above. We identified 1,333 13-mers associated with the A subgenome, and 33,845 associated with the C subgenome. We aligned these 13-mers to the entire genome, and identified the same subgenome identities proposed by Song *et al*.[3] based on comparisons with diploid relatives.

We then used ANOVA plus Tukey's test to assess the ability of all 13–mers to cluster the A and C subgenomes as previously defined. We found 129 13-mers enriched in the A subgenome, and 88,071 13-mers enriched in the C-subgenome. We show the clustering of chromosomes in Supplementary Figure 2a, using a subset of 129 random 13-mers from the C-enriched set in order to show both equally.

Supplementary Note 2. Identification of *Gossypium* subgenome-specific 13-mers

We used Jellyfish[1] to count the 13-mers of the tetraploid *Gossypium hirsutum* (AD1) and *Gossypium barbadense* (AD2) genome sequences[4]. Each of the tetraploid cotton genomes (2*n*=4*x*=52) are divided into *x*=13 sets of homoeologous pairs.

For each cotton genome, we computed the 13-mers that were (1) found in at least 100 copies in the entire tetraploid genome, and (2) at least two-fold enriched in one of the chromosomes in a

pair, without regard to the previous subgenome assignments[4]. For AD1, we found 101,837 13-mers enriched on the A subgenome, and 25,795 13-mers enriched on the D subgenome. For AD2, we found 100,832 on A, 26,827 on D. 85,422 A 13-mers and 20,518 D 13-mers are shared between both species. These 13-mers consistently grouped chromosomes into the A and D subgenomes that have been previously defined.

We then tested all 13-mers for their ability to separate the two clusters of chromosomes by ANOVA plus Tukey's Range Test (using aov and TukeyHSD functions in base R). We found 320,481 13-mers enriched in A relative to D of AD1, and 311,195 13-mers enriched in AD2. 276,265 of these overlapped between the two genomes. Similarly there are 94,305 enriched in D relative to A in AD1, and 86,379 enriched in D relative to A in AD2 (Figure 3 a-c; Supplementary Figure 3a-c;Supplementary Data 7-8). 71,988 of these overlapped between the two genomes. The increased number of enriched 13-mers in the ANOVA/Tukey analysis relative to the 2-fold enriched set described in the previous paragraph is due to a large number of 13-mers that significantly differentiate the subgenomes but have less than 2-fold enrichment. These include many commonly occurring 13-mers (occurring hundreds of times in each chromosome) that have only small differences between subgenomes. In order to focus on the substantial differences, we visualize only 13-mers that show a 100x bias in Figure 3d and Supplementary Figure 3d.

The large number of shared subgenome-enriched 13-mers between species is consistent with the model that these two allotetraploids are derived from the same polyploidization event. The 13-mers counts differ between the two cotton genomes because of differences in assembly methodology and quality and/or differences in residual transposon activity after speciation. Distinguishing between these two potential sources of differences is beyond the scope of this paper.

Supplementary Note 3. Identification of cyprinid subgenome-specific 13-mers

We used Jellyfish[1] to count the 13-mers of the tetraploid goldfish[5] and Hebao red carp[6] genome sequences. Both of the tetraploid cyprinid genomes ($2n=4x=100$) are divided into x=25 sets of

homoeologous pairs. Each pair corresponds to a single chromosome of diploid cyprinids with *n*=25.

For each cyprinid genome, we computed the 13-mers that were (1) found in at least 100 copies in the entire tetraploid genome, and (2) at least two-fold enriched in one of the chromosomes in a pair, without regard to the previous subgenome assignments. To be considered further, a 13-mer had to be enriched in this manner in 24 out of 25 pairs of chromosomes. This condition arises because goldfish chromosome 33 (GF33) was found to be an outlier with a low number of assembled repeats or proteins and was therefore left out of the initial identification of 13-mers. These subgenome enriched 13-mers consistently grouped chromosomes into subgenomes, including GF8, the homoeolog of GF33, and CC16/CC15, the orthologs of the goldfish pair mentioned above. We then tested all 13-mers for their ability to separate the two clusters of chromosomes by ANOVA plus Tukey's Range Test (using aov and TukeyHSD functions in base R). We found 1,867 13-mers enriched in the A-subgenome relative to B carp, and 189 13-mers enriched in the corresponding P-subgenome relative to M in goldfish. (Here A/B and P/M are the names given to subgenomes by the carp and goldfish papers, respectively; A corresponds to P and B corresponds to M). 185 of these overlapped between the two genomes. Similarly, there are 6,542 enriched in B relative to A in carp, and 832 enriched in B(=M) relative to A(=P) in goldfish (Supplementary Figures 3-4; Supplementary Data 9-10). 822 of these overlapped between the two genomes. The excess of subgenome-enriched 13-mers identified in carp is likely due to the better quality of the genome assembly.

The 5' end of GF4 and the 3' end of CC8 are orthologous (the chromosomes are assembled in the opposite orientation) and share a reduction in repetitive signal of all types, which has been noted in ribosomal DNA regions in other polyploids[7,8]. The ribosomal RNA genes are not placed on chromosomes in the current goldfish and common carp assemblies but are found on scaffolds in both species. Whether this low-repeat-density region is due to ribosomal DNA that is not assembled well in the chromosome, or some other chromosome substructure requires deeper cytogenetic investigation into the localization of ribosomal genes in the cyprinid genomes.

Supplementary Note 4. Identification of Camelina sativa subgenome-specific 13-mers

We used Jellyfish[1] to count the 13-mers of the allohexaploid *C. sativa* genome sequence[9]. Due to the chromosomal rearrangements in *C. sativa* documented by Kagale *et al.*[9], we initially identified candidate subgenome-enriched 13-mers by considering three clearly paralogous chromosome trios ((Csa15, Csa19, Csa01); (Csa17, Csa14, Csa3); and (Csa04, Csa06, Cs09)). We computed the 13-mers that were (1) found in at least 100 copies in the entire hexaploid genome, and (2) at least two-fold enriched in one of the three members of each of the triplets mentioned above relative to the other members, without regard to the previous subgenome assignments[9,10]. These 13-mers were used to cluster all chromosomes, resulting in subgenomes that are identical to those identified by Chaudhary *et al.*[10] .

We then tested all 13-mers for their ability to separate the two clusters of chromosomes by ANOVA plus Tukey's Range Test (using the aov and TukeyHSD functions in base R). We found strong differentiation between SG3 and SG1 and SG2, but weaker differentiation between SG1 and SG2. Specifically, we found 2,783 13-mers that are systematically enriched on SG3 relative to SG1 and SG2, and conversely 714 13-mers that are enriched on SG1 and SG2 relative to SG3, but only 27 13-mers enriched exclusively on SG2 and 158 13-mers enriched exclusively on SG1 (Figure 4a-c; Supplementary Figure 6 h-i; Supplementary Data 11). The distribution of these k-mers along the *C. sativa* chromosomes shows the robustness of these markers and the manner in which they cover the chromosomes (Figure 4 d-e). We note that different types of transposable elements have different insertion preferences relative to telomeres and centromeres along the genome.

While a complete characterization of the transposon content of the *C. sativa* genome is beyond the scope of this paper. We ran LTRHarvest[11] to identify any LTR-retrotransposons that might overlap of subgenome-enriched 13-mers. LTRHarvest rapidly identifies intact LTR retrotransposons and importantly links 5' and 3' ends of each element, facilitating timing of retrotransposon insertion based on 5'/3' divergence as described below for strawberry. We annotated 82,044,704 bp (13.48% of the genome) of sequence as LTR retrotransposons using

default parameters. We used bedtools[12] in order to assess overlap of these sequences with the subgenome-enriched 13-mers (Supplementary Data 1).

Homoeologous regions near that near the 3’ ends of Csa16 and Csa7 have low density of sub-genome specific markers; we note that these regions are co-orthologous to a known ribosomal DNA region in *Arabidopsis*. This is reminiscent of the parallel finding in goldfish and common carp and suggests that these regions plausibly contain ribosomal DNA in *C. sativa* as well. While the SG2 k-mer density is low, the different sets of repeats we identify could be used to build a set of probes to efficiently scan the *Camelina* radiation for the diploid SG2 progenitor, providing an alternative to the comprehensive population genetic analysis of Chaudhary *et al*[10].

Supplementary Note 5. Identification of Strawberry subgenome-specific 13-mers

We used Jellyfish[1] to count the 13-mers of the octoploid *Fragaria* x *ananassa* genome sequence from Edger *et al*.[13] The octoploid strawberry genome ($2n$=$8x$=56) is divided into x=7 sets of four homoeologous quartets, with each quartet corresponding to a single chromosome of the diploid woodland strawberry *F. vesca* and the Japanese diploid *F. iinumae*[13–20].

We computed the 13-mers that were (1) found in at least 100 copies in the entire octoploid genome, and (2) at least two-fold enriched in one of the four members of each quartet relative to the other members, without regard to the subgenome assignment of Edger *et al*.[13] To be considered further, a 13-mer had to be enriched in this manner in all seven quartets. The diploid genomes were not involved in identifying these 13-mers. This computation defined 829 13-mers with potential subgenome contrasts. These 13-mers consistently grouped chromosomes into four subgenomes. In the notation of the main text and below, we found 488 13-mers enriched in I, T1, and T2 (relative to V); 175 enriched in V (relative to I, T1, and T2); 102 enriched in T1 and T2 (relative to I and V); 39 enriched in T1; and 25 enriched in I (Supplementary Data 12).
Figure 5a shows a hierarchical clustering of the 56 chromosome of *F.* x *ananassa* and the 7 chromosomes of each of the diploids *F. vesca* and *F. iinumae*, with distance function given by 1-r, where r is Pearson’s correlation of the 13-mer densities (normalized by chromosome length), using the “hclust” function in R. Chromosome clustering was insensitive to details (e.g., whether

or not the diploids were included), and the same groupings were also reproduced using other clustering methods. At the same time, the 13-mers themselves were also clustered using R, defining (in the notation of the main text and below) 13-mers enriched in the subgenome combinations V, I-T1-T2, T1-T2, I, and T1, as shown in Figure 5a.

We note that the 13-mers shown in Figure 5a are derived without regard to the diploids. Nevertheless, 13-mers that separate subgenomes of octoploid strawberry are also differentially enriched in diploids, indicating that the corresponding repetitive activity that differentiates the subgenomes of octoploid strawberry is shared with diploids. We emphasize that the clustering shown atop Figure 5a groups chromosomes by shared repetitive content and is not a phylogenetic tree. This clustering is consistent with prior findings that (1) the "V" subgenome of octoploid strawberry is closely related to *F. vesca* (2) the "I" subgenome is closely related to *F. iinumae*, and (3) *F. iinumae* is also related to the other two chromosome sets (identified here as T1 and T2 subgenomes). There are several potential explanations for the weaker 13-mer signal for the "V" and "I"-enriched 13-mers in diploid *F. vesca* and F. *iinumae*. First, the chromosomes of the diploids were only available in their hard-masked forms. This means that any 13-mers that overlapped with known repeat families in the diploids are not available to our analysis. Second, only the octoploid chromosomes were used to identify sub-genome enriched biased 13-mers. Thus, any divergence between the transposons could easily lead to a bias in identifying repeats more in the allo-octoploid than the diploids since they are not guaranteed to share the exact same transposon activity.

Our V and I subgenome assignments agree with previous assignments by Tennessen *et al.*[16], Edger *et al.*[13], and others [14,17–19] (summarized in ref. 21):

- V-subgenome. We identified the subgenome that groups with diploid *F. vesca* in Figure 5a as the "V" subgenome, consistent with Edger *et al.*[13] and previous work based on protein-coding gene phylogeny and similarity (summarized in ref. 21). The V subgenome is enriched for V-specific 13-mers includes chromosomes Fvb1-4, Fvb2-2, Fvb3-4, Fvb4-3, Fvb5-1, Fvb6-1, and Fvb7-2.
- I-subgenome. Of the three subgenomes that group with *F. iinumae* in Figure 5a, one of them is consistently described as the "I" subgenome by multiple authors, based on

protein-coding gene phylogeny and similarity[13,14,17] (see summary in ref. 21). The I subgenome is enriched for I-specific 13-mers, and also for 13-mers enriched in I-T1-T2. The I subgenome includes chromosomes Fvb1-2, Fvb2-4, Fvb3-2, Fvb4-4, Fvb5-3, Fvb6-3, and Fvb7-3.

The remaining two sets of chromosomes were recognized by Tennessen *et al*.[16] as (1) closer to each other than to I or V, and (2) closer to I than to V. They have been separated into two sub-genomes by several groups (summarized in ref. 21) typically based on their similarity to *F. iinumae*, which is recognized as a weak criterion (particularly if these two subgenomes are sister to one another, and so phylogenetically equidistant from *F. iinumae* as suggested by Tennessen *et al*.[16]). Edger *et al*.[13] partitioned these fourteen chromosomes into two sets based on their protein-coding similarity to *F. nipponica* and *F. viridis*, but this was called into question by Liston *et al*.[14].

We find a well-supported alternate subgenome partition into T1 and T2 subgenomes that is distinct from previous studies:

- The T1-subgenome. The "T1" subgenome is enriched for T1-specific 13-mers, and also for 13-mers enriched in I-T1-T2 and T1-T2. It is composed of chromosomes identified by Edger *et al*. as belonging to the hypothesized 'nipponica' or 'viridis' subgenomes based on protein-coding similarity and phylogenetic analysis in comparisons with these and other diploids. The T1-subgenome includes chromosomes Fvb1-1, Fvb2-1, Fvb3-1, Fvb4-1, Fvb5-4, Fvb6-2, and Fvb7-4.
- The T2-subgenome. The "T2" subgenome is complementary to T1. It is marked by 13-mers enriched in I-T1-T2 and T1-T2 but not the T1-specific 13-mers. It is composed of chromosomes identified by Edger *et al*.[13] as belonging to the "nipponica" or "viridis" subgenomes based on protein-coding similarity and phylogenetic analysis in comparisons with these and other diploids. The T2 subgenome includes chromosomes Fvb1-3, Fvb2-3, Fvb3-3, Fvb4-2, Fvb5-2, Fvb6-4, and Fvb7-1.

Figure 5c-e show the distribution of V, I, T1, T1-T2, and I-T1-T2 13-mers across each chromosome. In each karyogram the chromosomes are shown in the order V, I, T1, and T2. There are occasional concentrations of unexpected 13-mers, e.g., the V-enriched sequence at the

3’ end of Fvb1-1 (which is otherwise assigned to the T1 subgenome). Since the 13-mers are produced by transposon activity, they mark the chromosome identity at the time of transposon insertion. Segments with anomalous 13-mers correspond to homoeologous exchanges.

We note that although *F.* x *ananassa* is a hybrid of two octoploids, *F. chiloensis* and *F. virginana*, these two North American species diverged after octoploid formation, and are interfertile, as demonstrated by the conventional disomic meiotic map produced by Hardigan *et al.*[21]. Thus, we expect their subgenome structure to be the same.

Supplementary Note 6. Analysis of variance for subgenome partitions

We assessed the significance of different subgenome partitions of the *F.* x *ananassa* genome using analysis of variance (ANOVA), considering the normalized counts per chromosome of the 423,429 13-mers that occur at least 100 times in the octoploid genome. We adopted a significance threshold of 0.05; after Bonferroni correction the threshold becomes $p < 10^{-7}$. We find that 92 13-mers support our T1-T2 subgenome partition, with 91 found more often on T1 than T2, and 1 found more often on T2 than T1. Similarly, 545 13-mers support the partition of I relative to T1 or T2 (taking the unique list combining I-T1 and I-T2), and 4,020 13-mers support the partition of the V subgenome from I, T1, or T2.

Figure 5b shows that the T1-specific 13-mers (black circles) are significant in our clustering (as expected based on their definition). All 13-mers that occur at least 100 times in the octoploid genome are shown, with 13-mers identified from our two-fold-enrichment-across-all-quartets criterion shown in color and others in gray. Evidently there are additional 13-mers with significant T1-T2 contrasts that did not meet the stringent two-fold enrichment criterion imposed in Supplementary Note 5. In contrast, we find no 13-mers that are significant in the ‘*nipponica’*-*’viridis’* grouping.

Our statistical framework allows us to test whether the proposed ‘nipponica’ and ‘viridis’ subgenomes Edger *et al.*[13], are consistent with the 13-mer counts per chromosome. We performed ANOVA, using both our grouping of chromosomes into subgenomes (V, I, T1, T2) and Edger *et al.*’s proposed (V, I, ‘nipponica’, ‘viridis’) subgenomes. Since we agree on the V

and I subgenomes, we expect V-specific, I/-T1/-T2-specific, and T1/-T2-specific 13mers to support both clusterings. Note that T1 and T2 together comprise the same 14 chromosomes as ‘nipponica’ plus ‘viridis’, so any 13-mer contrasts between these chromosomes and V and I do not provide support for either of the two hypotheses. The T1 vs. T2 specific 13mers, however, contradict the *nipponica-viridis* clustering of Edger *et al.*[13] (Figure 5a; Supplementary Figure 7a). We performed a Tukey’s range test (implemented in R with the TukeyHSD function to assign statistical significance to each pairwise subgenome comparison for each 13-mer (Figure 5b; Supplementary Figure 6b). We found significant (p<1e-6) differences between all pairwise subgenome comparisons except *‘nipponica’-’viridis’*. We include results comparing the proposed *‘nipponica’-’virdis’* subgenomes to I-subgenome in Supplementary Figure 6 in order to show that 13-mers that differentiate I from T1/T2 are still present in the *‘nipponica’-’viridis’* clustering, but specifically there are no 13-mers that differentiate the two controversial subgenomes.

In addition to the parametric analysis, we performed a Dunn’s test as a non-parametric way of identifying enrichment of 13-mers in subgenomes (Supplementary Figure 8). The non-parametric analysis reveals fewer significant 13-mers, but again supports our subgenome clustering.

Supplementary Note 7. Timing of strawberry retrotransposon activity and polyploidy

In order to infer the timing of subgenome-associated transposon activity, we identified LTR retrotransposons in the *F.* x *ananassa* genome using LTRHarvest[11]. We found subgenome-specific LTR retrotransposons by overlapping these LTR-annotated sequences with the subgenome-enriched 13-mers defined in Supplementary Note 5, and defined families of subgenome-specific LTRs by sequence-based clustering (using alignments with at least 90% length of the longer sequence and 1e-2 e-value cutoff) using all-vs-all BLASTN[22] with all other parameters set to their default values. Since the 5’ and 3’ long terminal repeats (LTRs) are identical at the time of insertion, the sequence divergence of intact 5’/3’ pairs is proportional to the time since insertion[23–25]. We measured 5’-3’ sequence divergence by Jukes-Cantor distance using the ape package in R[26].

In order to calibrate 5'-3' LTR sequence divergence to geological time, we reasoned that best hits of LTRs from the diploid *F. vesca* to LTRs of the I-T1-T2 subgenomes of octoploid *F. x ananassa* would represent divergence of ancient LTRs found in the last common ancestor of these genomes, circa 8 mya at the base of the *Fragaria* radiation[27]. As shown in Supplementary Figure 7, this distribution peaks at a Jukes-Cantor distance of ~0.11, so we assign the rate of nucleotide substitution in strawberry LTRs to be = 0.11/(2*8 million years) = 0.7×10^{-8} subs/year, or 1.4×10^{-8} for the 3.9 million year calibration. We note that the latter approximates the canonical value of 1.3×10^{-8} /yr used by Ma and Bennetzen[24] and San Miguel *et al*[25] for use with LTRs of grasses and often used more generally for plants including strawberry[13]. This canonical rate is derived from aligned intergenic regions, which according to Ma *et al*.[24] evolve twice as fast as silent sites in coding regions. Thus, our LTR calibration is consistent with rates found in other grasses.

Figure 5g shows 5'-3' LTR distances for LTR families with (1) at least 20 members and (2) overlap with at least one I-T1-T2-enriched 13-mer. Based on these 13-mers we infer that the LTR retrotransposons were active when the I-T1-T2 subgenomes were present in the same nucleus. Using our calibration, the peak of I-T1-T2 specific activity at 0.035 substitutions shown in Figure 5g corresponds to ~3 million years. We note that I-T1-T2-enriched families could also have been active in a last common ancestor of the I-T1-T2 progenitors. We consider this unlikely because (1) based on trees Liston *et al*. and Feng *et al*.[14,17] it is likely that the divergence of these progenitors was closer to the root of *Fragaria*, and (2) detectable 5'-3' LTR pairs are more common from recent activity, due to the ongoing mutation and loss of non-genic sequences in plant genomes[17].

Interestingly, we also find a recent uptick in activity (<0.01 subs ~ 1.5 mya) in these families that is present in both I-T1-T2 and V subgenomes, in roughly 3:1 proportion. We interpret this activity as arising from reactivation of I-T1-T2 transposons that were silenced in the hexaploid but released from silencing upon octoploid formation. The timing of this activity then roughly corresponds to the formation of octoploid, consistent with other timing estimates for this event based on protein-coding genes[13,28]. Finally, we also see a small peak in 5'-3' distance for I-T1-T2-type transposons on the V subgenome, roughly coincident with peak activity on I-T1-T2. We

interpret these LTR pairs as having originally been inserted on I-T1-T2 chromosomes during the hexaploid, but having ended up on the V subgenome due to subsequent homoeologous exchange in the octoploid. Note that the timing of homoeologous exchange does not affect the 5’-3’ LTR divergence, but merely transports the pair to another chromosome. A similar effect is evident in the calibration (Supplementary Figure 7b).

In a final note regarding timing, we note that in the read alignment based phylogenies of Liston *et al*.[14], the lengths of the “Camarosa” branches derived from the octoploid subgenomes are consistently longer than the lengths of their sister diploid *Fragaria* branches. Roughly the octoploid sequences are evolving ~60% faster than the diploids. This is to be expected due to the relaxation of purifying constraints in polyploids, due to redundancy[29,30]. Although we have identified T1 and T2 subgenomes based on repeat sequences that are different from the subgenomes inferred by Edger *et al*.[13], we have not positively identified the closest diploid lineages to T1 and T2. We agree with Tennessen *et al*., Liston *et al*., and Feng *et al*. that the T1-T2 subgenomes are closer related to *F. iinumae* but cannot be assigned more specifically to any extant diploid lineage[14,16,17].

Supplementary Note 8. Identification of *Nicotiana tabacum* subgenome-specific 13-mers and Hidden Markov Model

We used Jellyfish[1] to count the 13-mers of the *Nicotiana tabacum* chromosomes from assembly *N. tabacum* v1.0[31]. The tetraploid *N. tabacum* genome (2*n*=4*x*=48) are divided into two subgenomes with x=12 chromosomes.

We first used the protein-coding gene mapping Edwards *et al*.[31] to identify chromosome pairs that contained few to no rearrangements. Specifically, we used Nt01-Nt23, Nt16-Nt12, Nt6-Nt4, Nt10-Nt2, Nt11-Nt13, and Nt18-Nt9 as the initial search pairs. We computed the 13-mers that were (1) found in at least 100 copies in the entire tetraploid genome, and (2) at least two-fold enriched in one of the chromosomes of the pairs listed above. We identified 108,697 13-mers that are associated with the *tomentosiformis*-derived subgenome (T subgenome), and 386,983 associated with the sylvestris-derived subgenome (S subgenome). We aligned these 13-mers to the genome, and confirmed the same subgenomes proposed by Edwards *et al*.[31] based on

comparison with diploid *N. tomentosiformis* and *N. sylvestris*.We then tested all 13-mers for their ability to separate the two clusters of chromosomes by ANOVA plus Tukey's Range Test (using aov and TukeyHSD functions in base R[26]). We found 11,655 13-mers enriched in the T subgenome, and 13,447 13-mers enriched in the S subgenome. This reduction in total enriched 13-mers is likely due to the large amount of post-hybridization rearrangements that followed the genome duplication.

The hierarchical clustering of the tobacco chromosomes (Supplementary Figure 9a) shows weaker differentiation for Nt17, Nt18, Nt21, and Nt22. This is similar to results we observed in the *Miscanthus* genome on chromosomes that had homoeologous exchange[32]. Edwards *et al*.[31] note that these chromosomes also have regions that are the best hit to reads from both diploids, *N. tomentosiformis* and *N. sylvestris*. In order to ask if 13-mers could be used to identify the regions of homoeologous exchange, we used a Hidden Markov Model (HMM) using the post-Tukey 13-mer sets (Supplementary Figure 9e). To execute the HMM, we split the genome into 100-kb bins, and computed the density of S-enriched and T-enriched 13-mers in each bin. We used the Nt02-Nt10 pair to train the HMM as they had the fewest rearrangements in our map (Supplementary Figure 9d). We used equal T and S starting probabilities for each chromosome, a 99% self transition probability (T->T or S->S), and 1% between-subgenome probability (T->S and S->T). The only possible true states were S and T for the two subgenomes. Bins where log10(T-mer bp/S-mer bp) > 0.5 emitted "t", log10(T-mer bp/S-mer bp) < -0.5 emitted "s", and those in between were characterized as "unknown" subgenome. Using these criteria, the following values were used to generate an emission matrix for the training chromosomes. Nt10 emitted s, t, and unknown signals at a rate of 0.607573150, 0.2581756, and 0.3666093 respectively. Nt02 emitted s, t, and unknown signals at a rate of 0.004557885, 0.8467821, and 0.1467639 respectively.

Using the HMM package in R[32], we applied the above criteria to determine the Viterbi path of each chromosome, visualized in Supplementary Figure 9e.

Supplementary Note 9. Attempt to identify *Arabidopsis suecica* subgenomes

We used Jellyfish[1] to count the 13-mers of the *Arabidopsis suecica* chromosomes from assembly ASM1920280v1[33]. The tetraploid *A. suecica* genome is descended from a hybridization between an *A. thaliana*-like diploid ancestor (2*n*=10) and an *A. arenosa*-like autotetraploid ancestor (2*n*=16).

We partitioned the *A. suecica* chromosomes into three homoeologous groups based on the intra-genomic synteny mapping of Burns *et al*.[33]. Specifically (Chr1l Chr6/Chr7), (Chr2/Chr3, Chr8/Chr9/Chr10), and (Chr4/Chr5, Chr11/Chr12/Chr3) were used to define sub-genome 13-mer contrasts. 13-mer counts were summed and normalized by the total length of the chromosome set for this analysis. We computed the 13-mers that were (1) found in at least 100 copies in the entire tetraploid genome, and (2) at least two-fold enriched in one of the chromosomes of the pairs listed above. We found 36 13-mers that were enriched on the *A. thaliana*-like chromosomes, and 6,059 that were enriched on *A. arenosa*-like chromosomes via these criteria (Supplementary Figure 10a). We sample 36 *A. thaliana*-like 13-mers in order to balance the signal from each subgenome. Unlike many other tetraploids studied here, the subgenome-enriched13-mers are often found at high copy number on both subgenomes, but still asymmetrically distributed. Hierarchical chromosome clustering using these two-fold enriched k-mers correctly partitions the *A. suecica* genome into thaliana- and arenosa-like subgenomes.

When performing ANOVA+Tukey's Range test on the *A. suecica* data, however, we did not find any 13-mers that were statistically significant in differentiating the two subgenomes. Given the weak differentiation we observed for the asymmetric 13-mers in Supplementary Figure 10a this is likely due to the within group variance being similar to the between group variance. Thus, we find that while individually 13-mers do not show significant enrichment, collectively the entire set provides robust separation of subgenomes. This suggests that our k-mer-by-k-mer statistical approach is overly conservative, and more robust statistical methods could be developed.

Supplementary Note 10. Attempt to identify *Brassica rapa* subgenomes

For *Brassica rapa*, there have been a number of rearrangements since the last genome duplication event. Using the *Brassica rapa* cv. Chiifu V3.0 assembly[34], we extracted the

genomic blocks assigned to subgenomes by Zhang *et al*. based on biased fractionation (i.e., protein-coding gene retention rates) and annotated by them as corresponding to low fractionation (LF), medium fractionation 1 (MF1) and medium fractionation 2 (MF2) subgenomes. We used Jellyfish[1] to count 13-mers in these segments, and used ANOVA plus Tukey to assess if any 13-mers could differentiate the subgenomes as defined by Zhang *et al*.[34]. We found no 13-mers that supported this clustering. We tried clustering based on 13-mers that differentiated any segment, but no consistent clustering was observed (Supplementary Figure 10b).

In order to search for 13-mers that supported *any* clustering of chromosomes, we first considered homoeologous segments corresponding to ancestral *Brassica* elements F, J, R, and U, as these were the longest blocks of synteny. (This is parallel to the use of the three triples of chromosomes in *C. sativa*.) We found no 13-mers that differentiated *B. rapa* subgenomes. This is not surprising as the oldest repetitive elements in the *B. rapa* genome are around 6 million years old[34], and the divergence of *B. rapa* subgenomes is estimated to be substantially older than that[35]. Thus, relicts of progenitor-specific transposon activity has likely been erased by subsequent mutation and genomic turnover.

Supplementary Table 1. Genomes used for analysis.

**Species**	**Genome version**	**Source**
Fragaria x *ananassa*	*Fragaria* x *ananassa* Camarosa Genome v1.0	https://www.rosaceae.org/
Fragaria vesca	v4.0	https://www.rosaceae.org/
Fragaria iinumae	v1.0	https://www.rosaceae.org/
Camelina sativa	v2.0	http://cruciferseq.ca/?q=node/16
Carassius auratus	carAur01	https://research.nhgri.nih.gov/goldfish/download.shtml
Cyprinus carpio 'wuyuanensis'	Hebao red carp May 2017*	https://ngdc.cncb.ac.cn/gwh/Genome/81/show
Brassica napus	*Brassica_napus* cv. ZS11 PacBio V1.0	http://brassicadb.cn/
Brassica rapa	*Brassica rapa* cv. Chiifu V3.0	http://brassicadb.cn/
Nicotiana tabacum	*N. tabacum* v1.0	https://solgenomics.net/organism/Nicotiana_tabacum/genome
Arabidopsis suecica	ASM1920280v1	https://www.ebi.ac.uk/ena/browser/view/GCA_019202805.1
Gossypium hirsutum	NDM8	https://www.cottongen.org/data/download
Gossypium barbadense	Pima90	https://www.cottongen.org/data/download

*At the time of writing, the Hebao red carp chromosome-scale assembly is only available from the National Genome Database of China. Both NCBI and EBI have only contigs for this genome.

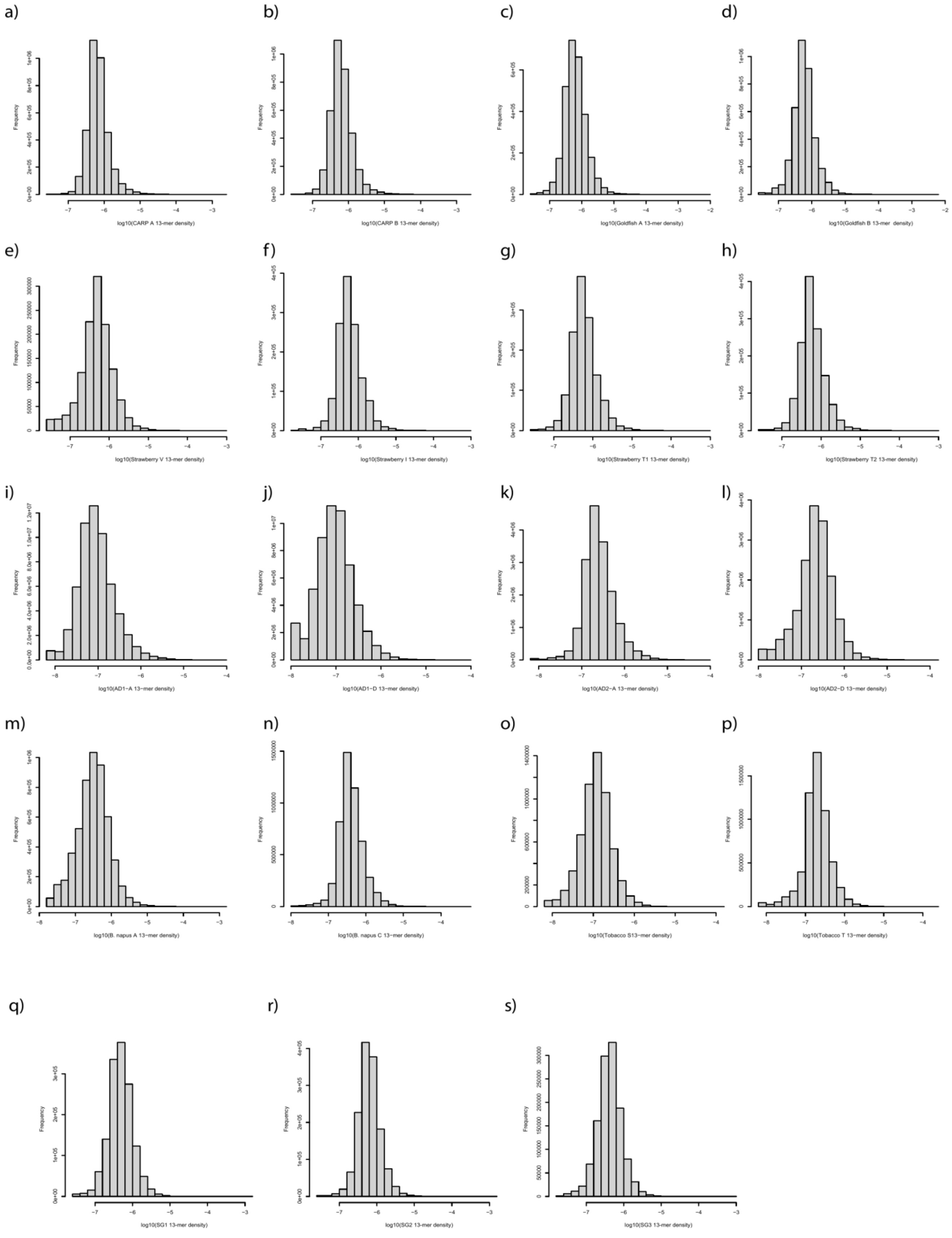

a)
b)
c)
d)
e)
f)
g)
h)
i)
j)
k)
l)
m)
n)
o)
p)
q)
r)
s)
Frequency
log10(CARP A 13-mer density)
log10(CARP B 13-mer density)
log10(Goldfish A 13-mer density)
log10(Goldfish B 13-mer density)
log10(Strawberry V 13-mer density)
log10(Strawberry I 13-mer density)
log10(Strawberry T1 13-mer density)
log10(Strawberry T2 13-mer density)
log10(AD1-A 13-mer density)
log10(AD1-D 13-mer density)
log10(AD2-A 13-mer density)
log10(AD2-D 13-mer density)
log10(B. napus A 13-mer density)
log10(B. napus C 13-mer density)
log10(Tobacco S13-mer density)
log10(Tobacco T 13-mer density)
log10(SG1 13-mer density)
log10(SG2 13-mer density)
log10(SG3 13-mer density)

Supplementary Figure 1. Histograms of subgenome 13-mer count/bp.

A requirement of ANOVA is that counts be approximately normally distributed **(a-s)** Histograms of the total 13-mer count/bp for each subgenome discussed in the paper, with species and sub-genome labels shown in x-axis labels. All appear approximately normal, satisfying the assumption of normality in ANOVA. Source data are provided as a Source Data file.

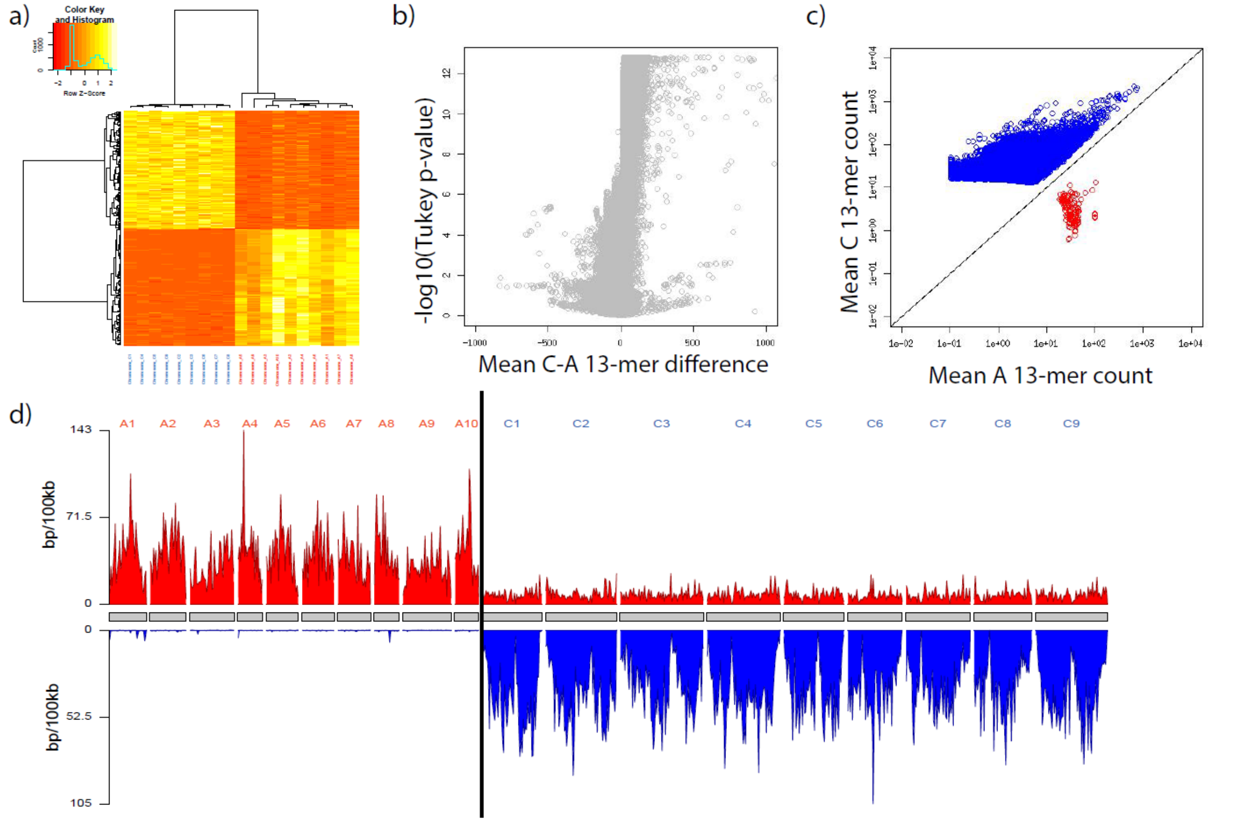

Supplementary Figure 2. ***Brassica napus*** **allotetraploidy.**

Brassica napus is an allotetraploid with AACC structure[2,3]. **a)** Heatmap showing *B. napus* chromosomes clustered based on 13-mers that differentiate pairs of homeologous chromosomes. Columns representing A- and C-subgenome chromosomes are labeled in blue and red, respectively. **b)** Volcano plot showing Bonferroni-corrected Tukey *p*-value vs. mean 13mer C-A count difference. Each point is a 13mer. **c)** Scatterplot showing mean A-vs-C subgenome 13-mer count/bp, with A- and C-enriched 13-mers shown in blue and red, respectively. Black line is y=x. **d)** Karyogram of A- and C-enriched 13-mer repeat density in 100 kb bins along each *B. napus* chromosome. A-enriched 13-mer density is shown above the line in blue, and B-enriched 13-mer density below in red. Subgenomes are separated by black vertical line. Source data are provided as a Source Data file.

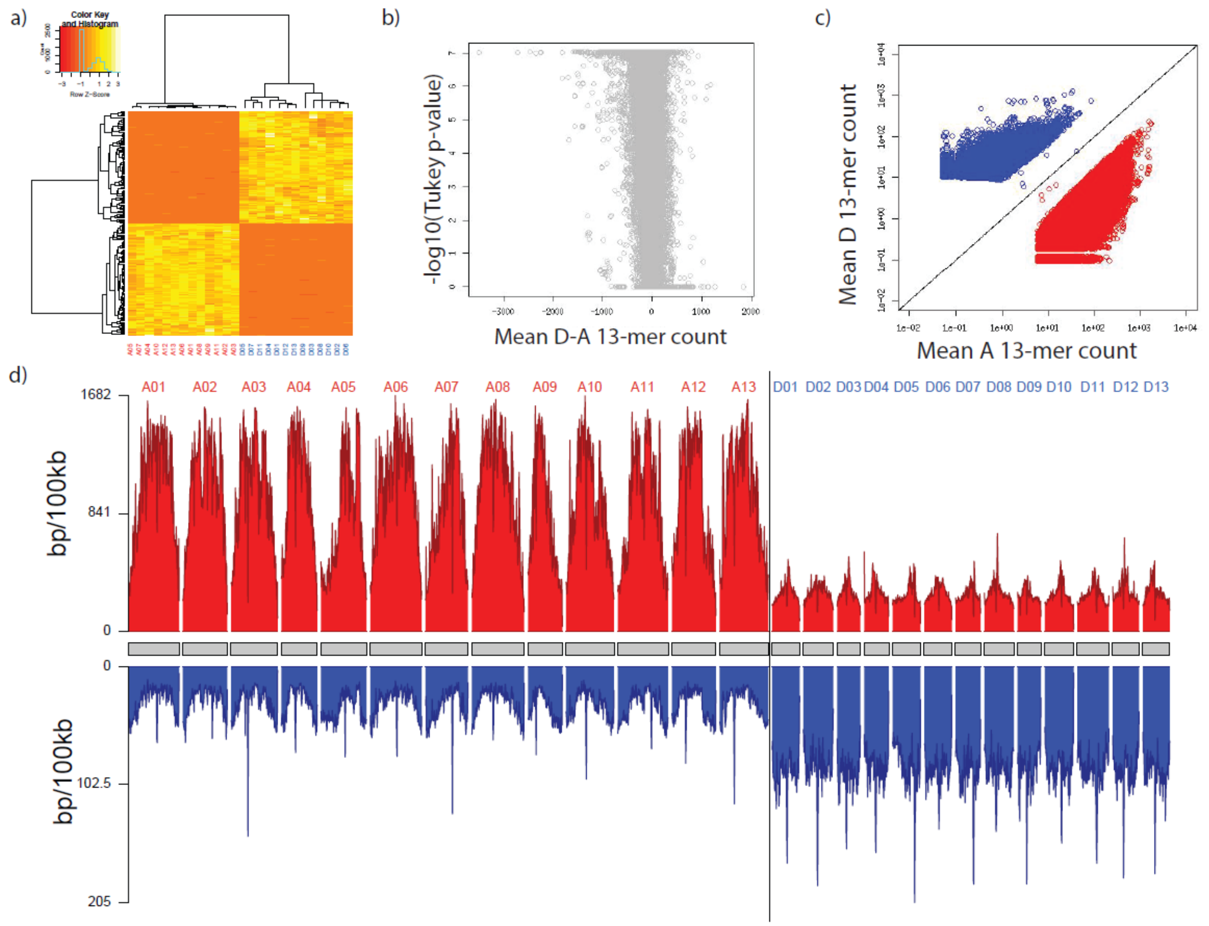

Supplementary Figure 3. *G. barbadense* (AD2) allotetraploidy.

Paralleling the analysis of upland cotton (*Gossypium hirsutum*) shown in Figure 3, here we show the corresponding analysis of the related *G. barbadense*, which is also an AADD allotetraploid, designated AD2[4] **a)** Heatmap showing *G. barbadense* chromosomes clustered based on 13-mers that differentiate pairs of homeologous chromosomes. Columns representing A- and D-subgenome chromosomes are labeled in blue and red, respectively. **b)** Volcano plot showing Bonferroni-corrected Tukey *p*-value vs. mean 13mer count difference D-A between subgenomes of *G. barbadense*. Each point is a 13mer. **c)** Scatterplot showing mean A-vs-D subgenome 13-mer count/bp, with A and D-enriched 13-mers shown in blue and red, respectively. Black line is y=x. **d)** Karyogram showing density of A-enriched (above, red) and D-enriched (below, blue) 13-mers in 100-kb bins along each of the chromosomes of *G. hirsutum*. (The densities shown here only count 13-mers with at least a 100x bias to avoid showing contributions from a few 13-mers that are weakly but statistically significantly enriched between subgenomes that lie near the equal line in panel c). Source data are provided as a Source Data file.

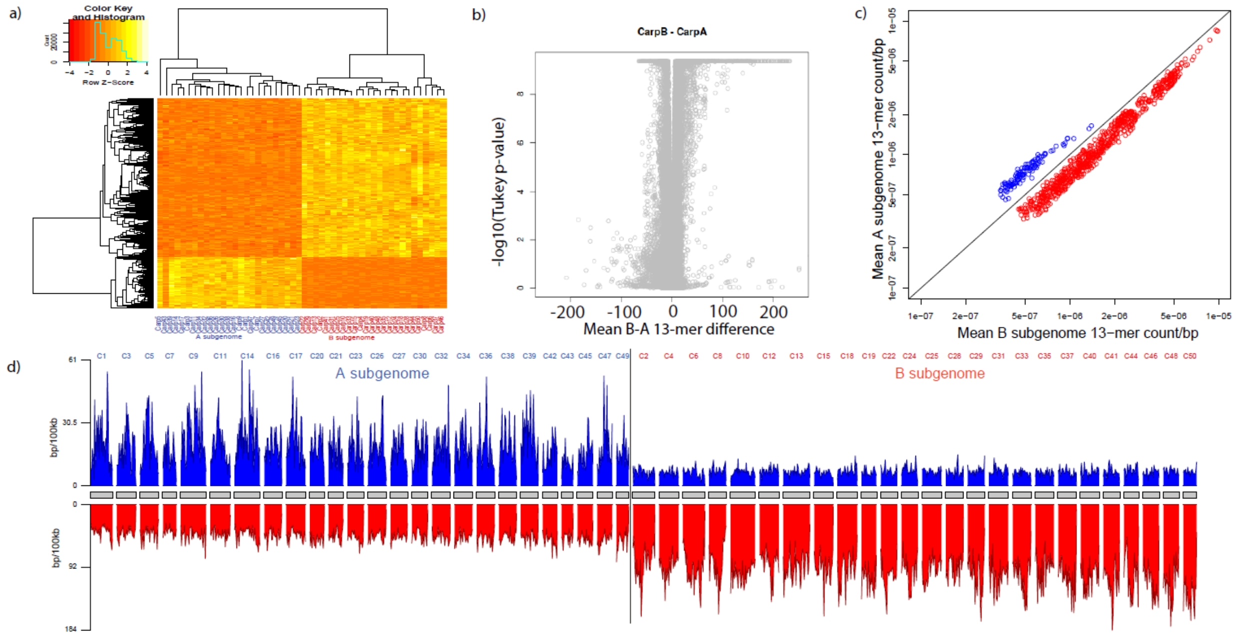

Supplementary Figure 4. Carp allotetraploidy.

Carp and goldfish are cyprinid fish that share allotetraploid origins[5,6] A parallel analysis for goldfish is shown in Supplementary Figure 5. **a)** Heatmap showing carp chromosomes clustered based on 13-mers that differentiate pairs of homeologous chromosomes. A/P/S- and B/M/L-subgenome chromosomes are labeled in blue and red, respectively. **b)** Volcano plot showing Bonferroni-corrected Tukey *p*-value vs. mean 13mer B-A count difference. Each point is a 13mer. **c)** Scatterplot showing mean A-vs-B subgenome 13-mer count/bp, with A- and B-enriched 13-mers shown in blue and red, respectively. Black line is y=x. **d)** Karyogram of A- and B-enriched 13-mer repeat density in 100-kb bins along each carp chromosome, with A-enriched 13-mer density is shown above the line in blue, and B-enriched 13-mer density below in red. Subgenomes are separated by black vertical line. Source data are provided as a Source Data file.

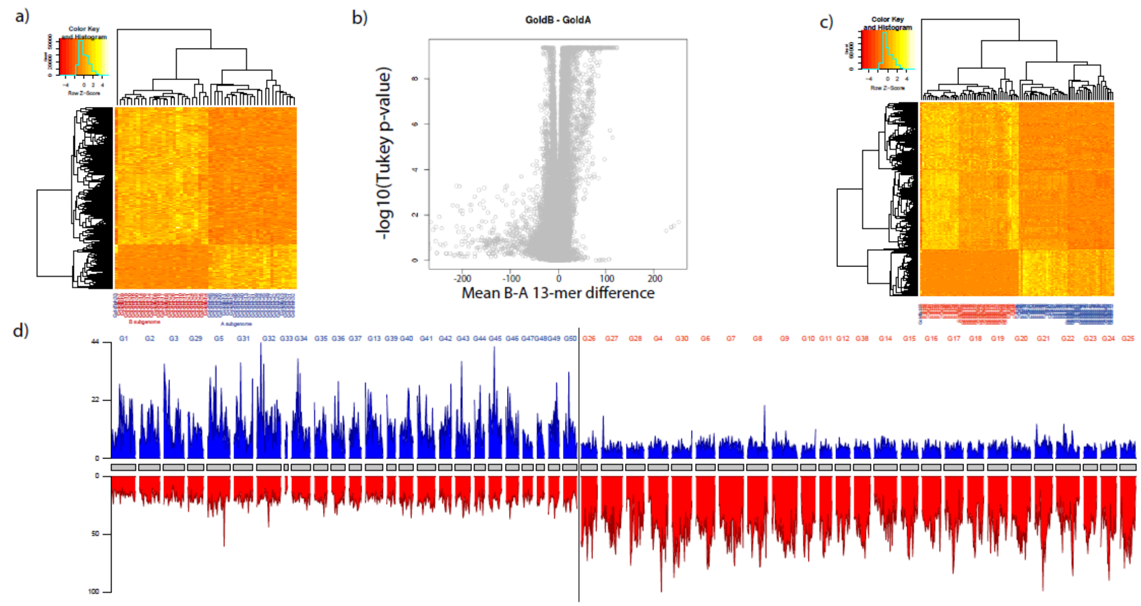

Supplementary Figure 5. Goldfish allotetraploidy.

Carp and goldfish are cyprinid fish that share allotetraploid origins[5,6] **a)** Heatmap showing goldfish chromosomes clustered based on 13-mers that are differentiate pairs of homeologous chromosomes. A/P/S- and B/M/L-subgenome chromosomes are labeled in blue and red, respectively. Goldfish33 is an outlier due to the problems in its assembly discussed in Cheng *et al*.[35] See parallel analysis for carp in Supplementary Figure 4. b) Volcano plot showing Bonferroni-corrected Tukey *p*-value vs. mean 13mer B-A count difference. Each point is a 13mer. **c)** Heatmap showing joint clustering of goldfish and carp chromosomes (columns) based on shared 13-mer content (rows). Note that goldfish and carp A/P/S-subgenome chromosomes cluster together (blue labels), as do B/M/L-subgenome chromosomes (red labels). Goldfish chromosome 33 is an outlier that does not cluster with either of the two chromosomal groups. The hierarchical clustering of chromosomes is a clustering based on shared repetitive content and does not indicate phylogenetic relationships. The shared repetitive signal between species is strong evidence they share the same allotetraploidy event. **d)** Karyogram of A and B 13-mer repeat density in 100-kb bins along each goldfish chromosome, with A-enriched 13-mer density shown above the line in blue, and B-enriched 13-mer density shown below in red. Subgenomes are separated by black vertical line. Source data are provided as a Source Data file.

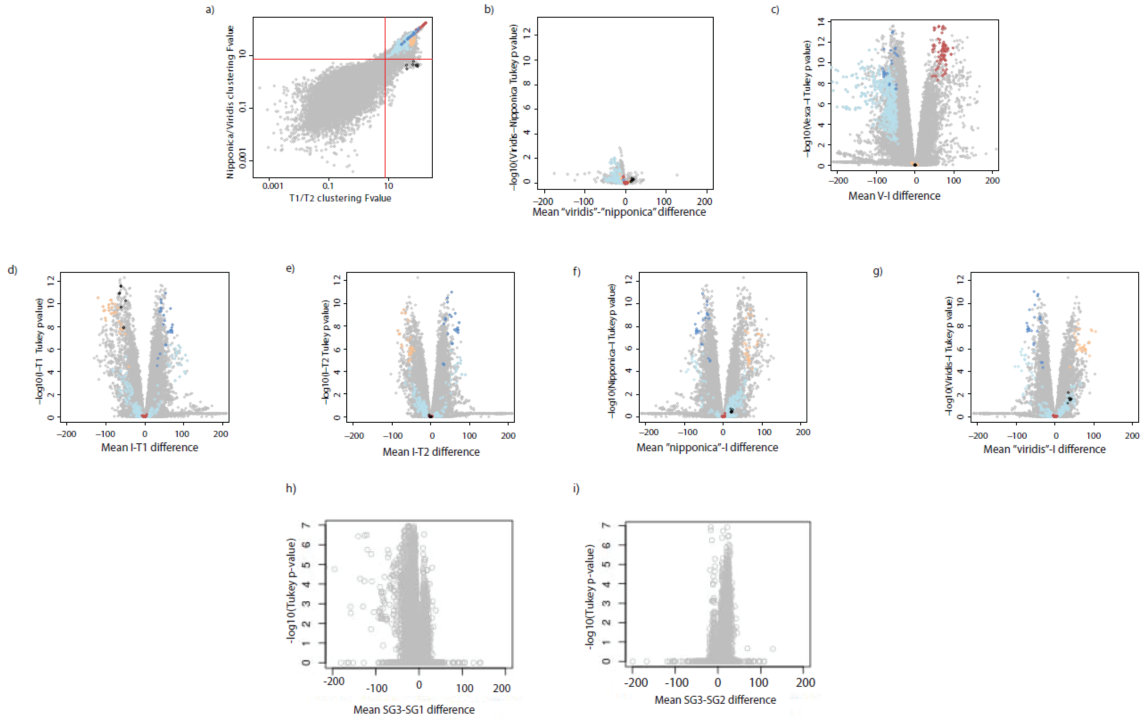

Supplementary Figure 6. Strawberry F value comparison, and strawberry and camelina volcano plots.
a) Comparison of ANOVA F value between the octoploid strawberry T1/T2 sub-genome partition proposed here (x-axis) and the 'nipponica-viridis' sub-genome partition proposed by Edger *et al*. [13] (y-axis). Red lines are drawn at F=7.55446, the significance value for p=0.001. Black dots (T1-13mers) are part of a larger cloud that passes the significance threshold for T1-T2 partition, but fails for the 'nipponica/viridis' partition. **b-f)** Volcano plots demonstrating significant differences in 13-mer content for pairwise subgenome comparisons: (b) V and I subgenomes, (c) I and T1, (d) I and T2, (e) I and 'nipponica', (f) I and 'viridis', where 'viridis' and 'nipponica' are defined in Edger *et al*. [13]. These latter two only show that I is different from both the 'nipponica' and 'viridis' chromosomes but do not have bearing on any differences between nipponica-and-viridis. Comparisons between V and other subgenomes are not shown, since V is highly differentiated relative to other chromosome sets regardless of partition **g)** 'Nipponica' and 'viridis' subgenomes as defined by Edger *et al* [13]. Compared with Figure 5b, there are no significant 13-mers that support this partition. **h,i)** Volcano plots for subgenome partitions of allohexaploid *Camelina sativa*, volcano plot showing support for SG3-SG1 (panel h) and SG3-SG2 (panel g). Volcano plot for SG1-SG2 is shown in Figure 4b. Source data are provided as a Source Data file.

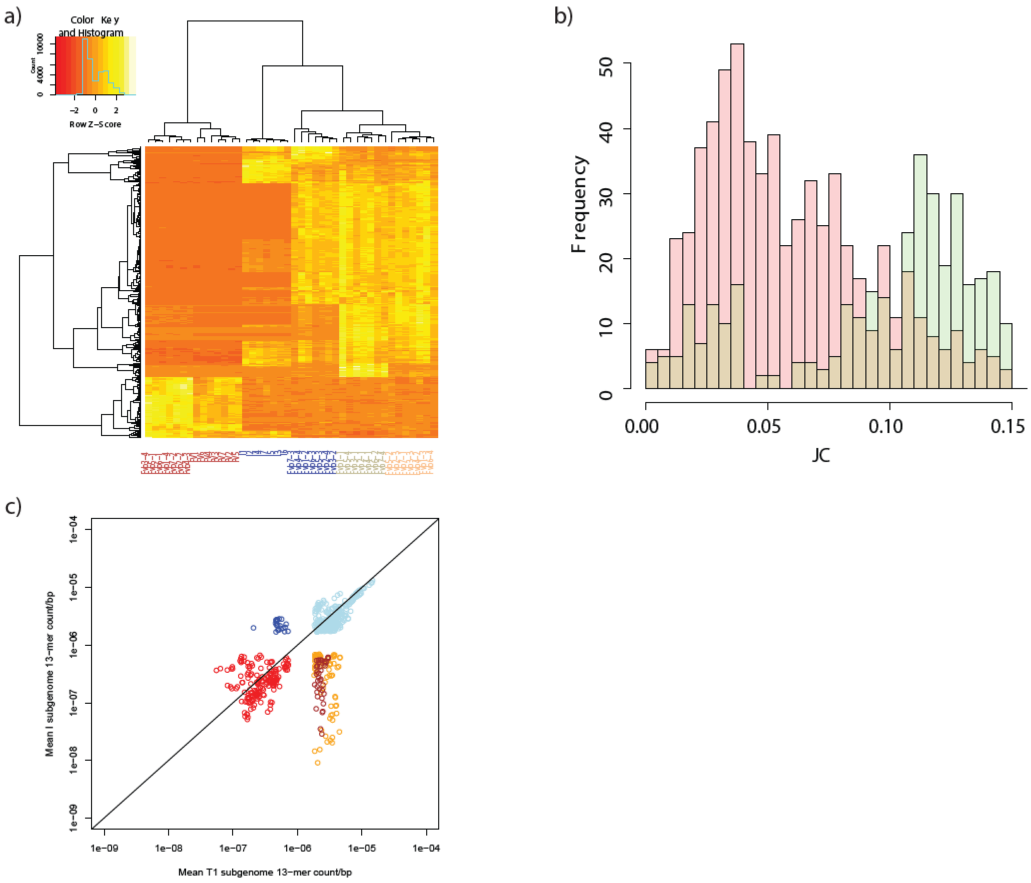

Supplementary Figure 7. Strawberry subgenome partitioning and divergence of subgenome-enriched LTR-retrotransposons.

Further support for strawberry subgenome partitioning. **a)** Hierarchical clustering of chromosomes based on the complete set of 829 repetitive 13-mers described in the text. (Figure 5a shows a subset.) V (F. vesca-like) chromosomes are in red, I (F. iinumae-like) chromosomes are in blue, T1 chromosomes in brown, T2 chromosomes in orange. Diploid vesca and iinumae chromosomes are included using FV and I as chromosome names with red and blue labels, respectively. The 13-mers were defined from the octoploid genome without reference to diploids, but are also shared by these other genomes, consistent with shared repetitive content. **b)** Histograms of Jukes-Cantor (JC) distance between long terminal repeats (LTRs) of diploid *F. vesca* and octoploid *F.* x *ananassa*, separated by subgenome type. Mutual best hits between all diploid *F. vesca* LTRs and all I-T1-T2 subgenome LTRs (green) peak at ~0.11, which we calibrate to 8 million years, i.e., the base of the Fragaria radiation. Mutual best hits between diploid *F. vesca* LTRs and the V subgenome peak more recently ~0.035, consistent with the close relationship between diploid *F. vesca* and this subgenome of octoploid strawberry. There is also a small recent green peak ~0.035, representing likely homoeologous exchange or recent activity after octoploid formation (that is, these elements were born on the V subgenome but ended up on I-T1-T2 chromosomes). **c)** Scatterplot showing mean T1 subgenome 13-mer count/bp on the x-axis, mean I subgenome 13-mer count/bp count on the y-axis. Black line shows y=x. Subgenome markers follow the same color conFigureuration as Figure 5. Source data are provided as a Source Data file.

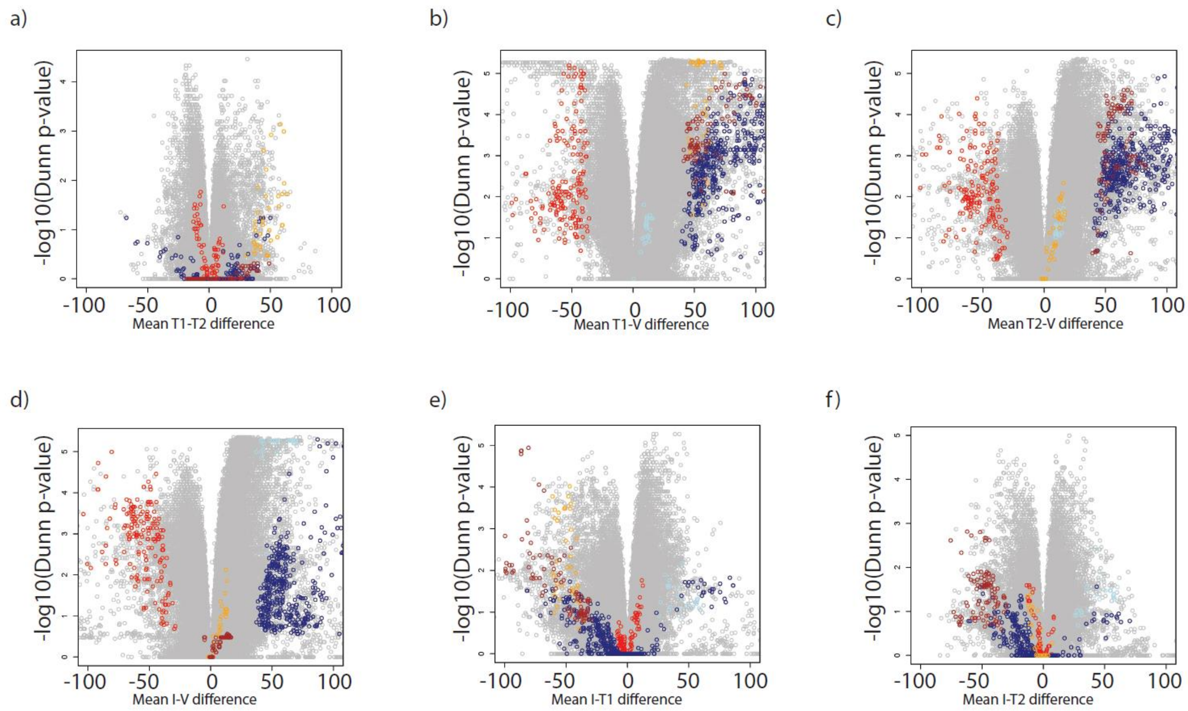

Supplementary Figure 8. Strawberry non-parametric volcano plots.

Non-parametric versions of the octoploid strawberry sub-genome contrasts shown in Figure 5b and Supplementary Figure 6. (a-f) Volcano plots showing the mean subgenome difference on x-axis, -log10(Dunn's *p*-value) on the y-axis. Colors of dots correspond with Figure 5. Source data are provided as a Source Data file.

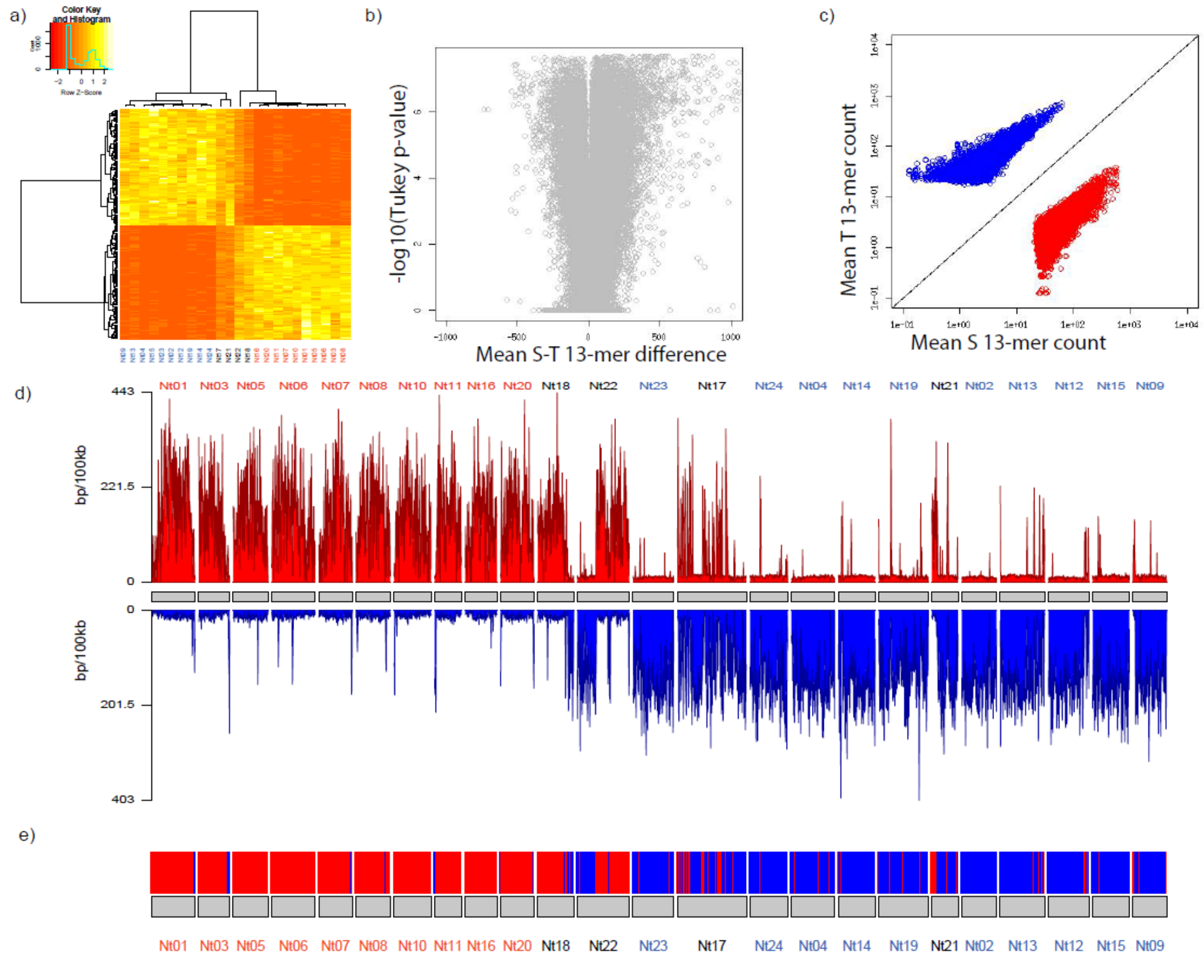

Supplementary Figure 9. *Nicotiana tabacum* allotetraploidy and Hidden Markov Model. *Nicotiana tabacum* is an allotetraploid formed by hybridization of *N. sylvestris*-like and *N. tomentosiformis*-like progenitors contributing S and T subgenomes, respectively [31]. **a)** Heatmap showing clustering of *N. tabacum* chromosomes (columns) using a sample of 100 13-mers found to differentiate subgenomes (rows), with S chromosomes labeled in red and T chromosomes blue. Four chromosomes (Nt17, Nt21, and Nt18, Nt22) are clustered with T and S chromosome, respectively, but show reduced 13-mer signals. **b)** Volcano plot showing Bonferroni-corrected Tukey *p*-value vs. mean 13mer S-T count difference. Each point is a 13mer. **c)** Scatterplot showing mean count 13-mer counts on T vs S chromosomes. S and T-enriched 13-mers are shown in red and blue, respectively, and line is y=x. Only the 13-mers that significantly differentiate subgenomes are shown. **d**) Karyogram of S- and T-enriched 13-mers density in 100 kb bins along each allotetraploid tobacco chromosome, with S–enriched density shown above the line in red and T-enriched density shown below in blue. The pattern of subgenome-enriched 13-

mers is similar to the pattern of *N. sylvestris* and *N. tomentosiformis* diploid reads aligned to the tetraploid genome [31], suggesting that these 13-mers track the same rearrangements as the diploid reads. Note that chromosomes Nt18, Nt22, Nt17, and Nt21 (black labels) show mixed signal in discrete blocks along the chromosome. **e)** Segmentation of *N. tabacum* chromosomes using an Hidden Markov Model (HMM) to recognize S- and T-like chromosome segments, with 100 kb resolution. HMM was trained on the Nt02/Nt10 pair. The details of the HMM are discussed in Supplementary Note 8. Source data are provided as a Source Data file.

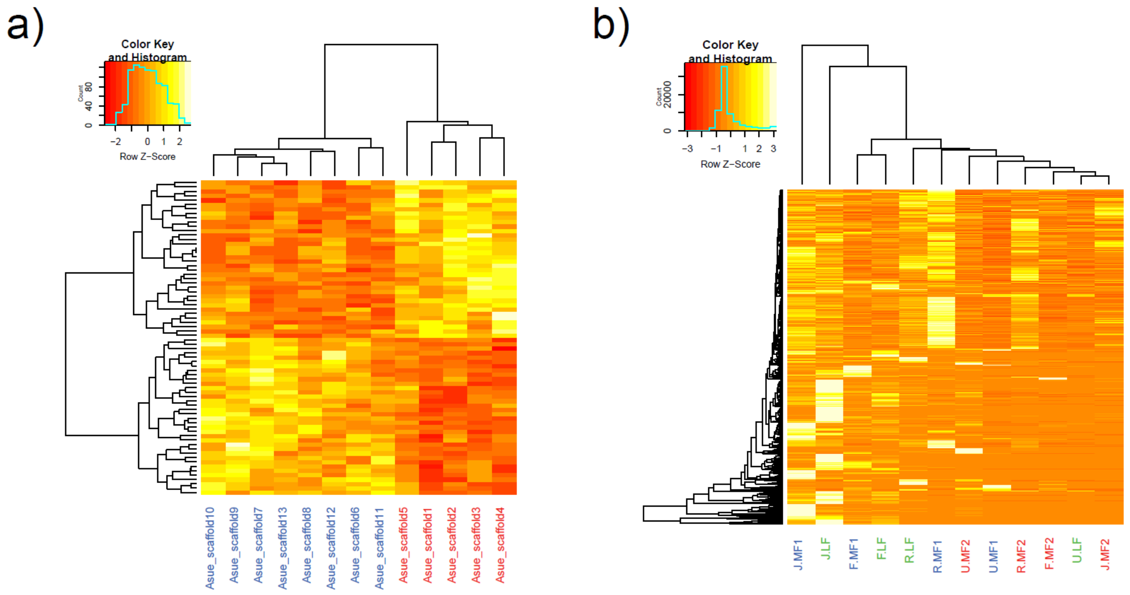

Supplementary Figure 10. *Arabidopsis suecica* and *Brassica rapa* allotetraploidies.
For polyploids formed from weakly diverged progenitors without clearly defined progenitor-specific repetitive content, or that arose too long ago for signatures of subgenome-specific transposable elements to persist in the genome due to accumulated mutation, the k-mer method described here may not clearly differentiate subgenomes. **a)** *Arabidopsis suecica* is an allotetraploid formed from the hybridization of *A. thaliana* and *A. arenosa*-like progenitors. Heatmap showing clustering of *A. suecica* chromosomes (rows) clustered based on 13-mers that differentiated subgenomes based on ratios between homoeologous chromosome pairs. *A. thaliana*-like chromosomes are labeled in red and *A. arenosa*-like chromosomes are labeled in blue[33]. While these 13-mers do differentiate subgenomes, there is a large amount of shared signal between subgenomes when compared to other tetraploids. **b)** *Brassica rapa* is an ancient allohexaploid that has experienced extensive rearrangements. Heatmap shows clustering of segmental homoeologous blocks identified by Zhang *et al*.[34] based on 13-mers found to differentiate any of the segments Segments are labeled MF1, MF2, LF (based on medium and how fractionation levels) that are believed to reflect subgenome identify. We identify no 13-mers in *B. rapa* that consistently cluster these segments in any combination, which could be due to decay of sub-genome-specific repetitive content by accumulated mutation and/or homoeoloogus rearrangements that mix subgenomes. Source data are provided as a Source Data file.

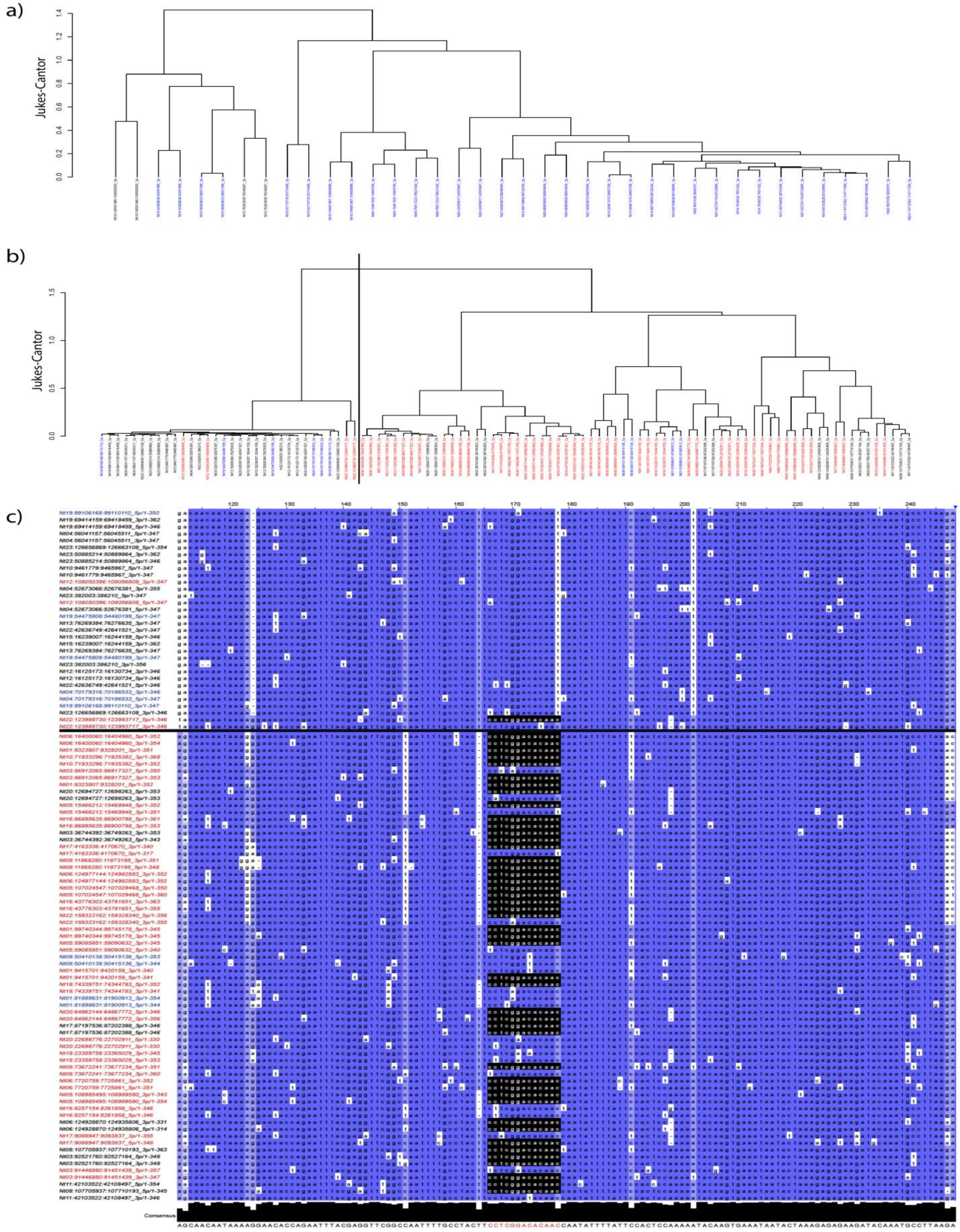
a)
b)
c)
Jukes-Cantor
Jukes-Cantor
Consensus

Supplementary Figure 11. Long-terminal repeat alignments of tobacco subfamilies that include diagnostic 13-mer.

a) Neighbor-joining tree of the long-terminal repeats (LTRs) of tobacco LTR-retrotransposon subfamily fam_150 using the Jukes-Cantor distance. Blue names indicate members marked by T-subgenome-enriched 13-mers. This family is exclusively found on T-subgenome segments, and many members overlap T-enriched 13-mers.

b) Neighbor-joining tree of the LTRs of tobacco retrotransposon subfamily fam_62 using Jukes-Cantor distance. Blue names indicate members marked by T-subgenome-enriched 13-mers. Red names indicate members marked by S-subgenome-enriched 13-mers. Vertical black line separates predominantly blue (T-subgenome-enriched) and red (S-subgenome-enriched) clades.

c) Part of a multiple sequence alignment of the LTRs of fam_62 above, which includes a S-subgenome-enriched 13-mer. The full fasta alignment is included in Supplementary Data 5. Black vertical line shows the break between the left and right clades of **(b)**. LTRs are colored and ordered top to bottom in the same way they are left to right above in **(b)**. The consensus sequence and graph are included on the bottom. The S-subgenome enriched 13-mer is shown in red in the consensus and is highlighted in black in the individual sequences. Dark blue background indicates bases that are at least 80% conserved, light blue bases are between 50% and 80%. Position 178 is C in the S-specific-clade but the T allele is found on both S and T subgenomes. Source data are provided as a Source Data file.

Supplementary references

1. Marçais, G. & Kingsford, C. A fast, lock-free approach for efficient parallel counting of occurrences of k-mers. *Bioinformatics* **27**, 764–770 (2011).
2. Chen, X. *et al.* A high-quality Brassica napus genome reveals expansion of transposable elements, subgenome evolution and disease resistance. *Plant Biotechnol. J.* **19**, 615–630 (2021).
3. Song, J.-M. *et al.* Eight high-quality genomes reveal pan-genome architecture and ecotype differentiation of *Brassica napus*. *Nat. Plants* **6**, 34–45 (2020).
4. Wang, M. *et al.* Reference genome sequences of two cultivated allotetraploid cottons, *Gossypium hirsutum* and *Gossypium barbadense*. *Nat. Genet.* **51**, 224–229 (2019).
5. Chen, Z. *et al.* De novo assembly of the goldfish (*Carassius auratus*) genome and the evolution of genes after whole-genome duplication. *Sci. Adv.* **5**, eaav0547 (2019).
6. Xu, P. *et al.* The allotetraploid origin and asymmetrical genome evolution of the common carp *Cyprinus carpio*. *Nat. Commun.* **10**, 4625 (2019).
7. Session, A. M. *et al.* Genome evolution in the allotetraploid frog *Xenopus laevis*. *Nature* **538**, 336–343 (2016).
8. Lovell, J. T. *et al.* Genomic mechanisms of climate adaptation in polyploid bioenergy switchgrass. *Nature* **590**, 438–444 (2021).
9. Kagale, S. *et al.* The emerging biofuel crop *Camelina sativa* retains a highly undifferentiated hexaploid genome structure. *Nat. Commun.* **5**, 3706 (2014).
10. Chaudhary, R. *et al.* Assessing diversity in the *Camelina* genus provides insights into the genome structure of *Camelina sativa*. *G3 Bethesda Md* **10**, 1297–1308 (2020).
11. Ellinghaus, D., Kurtz, S. & Willhoeft, U. LTRharvest, an efficient and flexible software for de novo detection of LTR retrotransposons. *BMC Bioinformatics* **9**, 18 (2008).

12. Quinlan, A. R. BEDTools: The swiss-army tool for genome feature analysis. *Curr. Protoc. Bioinforma.* **47**, 11.12.1-34 (2014).

13. Edger, P. P. *et al.* Origin and evolution of the octoploid strawberry genome. *Nat. Genet.* **51**, 541–547 (2019).

14. Liston, A. *et al.* Revisiting the origin of octoploid strawberry. *Nat. Genet.* **52**, 2–4 (2020).

15. Edger, P. P. *et al.* Reply to: Revisiting the origin of octoploid strawberry. *Nat. Genet.* **52**, 5–7 (2020).

16. Tennessen, J. A., Govindarajulu, R., Ashman, T.-L. & Liston, A. Evolutionary origins and dynamics of octoploid strawberry subgenomes revealed by dense targeted capture linkage maps. *Genome Biol. Evol.* **6**, 3295–3313 (2014).

17. Feng, C. *et al.* Tracing the diploid ancestry of the cultivated octoploid strawberry. *Mol. Biol. Evol.* **38**, 478–485 (2021).

18. Liu, B., Poulsen, E. G. & Davis, T. M. Insight into octoploid strawberry (*Fragaria*) subgenome composition revealed by GISH analysis of pentaploid hybrids. *Genome* **59**, 79–86 (2016).

19. Sargent, D. J. *et al.* HaploSNP affinities and linkage map positions illuminate subgenome composition in the octoploid, cultivated strawberry (*Fragaria×ananassa*). *Plant Sci. Int. J. Exp. Plant Biol.* **242**, 140–150 (2016).

20. Davik, J. *et al.* A ddRAD based linkage map of the cultivated strawberry, *Fragaria xananassa*. *PLoS ONE* **10**, e0137746 (2015).

21. Hardigan, M. A. *et al.* Genome synteny has been conserved among the octoploid progenitors of cultivated strawberry over millions of years of evolution. *Front. Plant Sci.* **10**, (2020).

22. Camacho, C. *et al.* BLAST+: architecture and applications. *BMC Bioinformatics* **10**, 421 (2009).

23. Dangel, A. W., Baker, B. J., Mendoza, A. R. & Yu, C. Y. Complement component C4 gene intron 9 as a phylogenetic marker for primates: long terminal repeats of the endogenous retrovirus ERV-K(C4) are a molecular clock of evolution. *Immunogenetics* **42**, 41–52 (1995).

24. Ma, J. & Bennetzen, J. L. Rapid recent growth and divergence of rice nuclear genomes. *Proc. Natl. Acad. Sci. U. S. A.* **101**, 12404–12410 (2004).

25. SanMiguel, P., Gaut, B. S., Tikhonov, A., Nakajima, Y. & Bennetzen, J. L. The paleontology of intergene retrotransposons of maize. *Nat. Genet.* **20**, 43–45 (1998).

26. R: The R Project for Statistical Computing. https://www.r-project.org/.

27. Qiao, Q. *et al.* Comparative transcriptomics of strawberries (*Fragaria* spp.) provides insights into evolutionary patterns. *Front. Plant Sci.* **7**, 1839 (2016).

28. Njuguna, W., Liston, A., Cronn, R., Ashman, T.-L. & Bassil, N. Insights into phylogeny, sex function and age of *Fragaria* based on whole chloroplast genome sequencing. *Mol. Phylogenet. Evol.* **66**, 17–29 (2013).

29. Lynch, M. The frailty of adaptive hypotheses for the origins of organismal complexity. *Proc. Natl. Acad. Sci. U. S. A.* **104 Suppl 1**, 8597–8604 (2007).

30. Force, A. *et al.* Preservation of duplicate genes by complementary, degenerative mutations. *Genetics* **151**, 1531–1545 (1999).

31. Edwards, K. D. *et al.* A reference genome for *Nicotiana tabacum* enables map-based cloning of homeologous loci implicated in nitrogen utilization efficiency. *BMC Genomics* **18**, 448 (2017).

32. Himmelmann, S. S.-D. L. HMM: Hidden Markov Models. (2022).
33. Burns, R. *et al.* Gradual evolution of allopolyploidy in *Arabidopsis suecica*. *Nat. Ecol. Evol.* **5**, 1367–1381 (2021).
34. Zhang, L. *et al.* Improved *Brassica rapa* reference genome by single-molecule sequencing and chromosome conformation capture technologies. *Hortic. Res.* **5**, 1–11 (2018).
35. Cheng, F. *et al.* Biased gene rractionation and dominant gene expression among the subgenomes of *Brassica rapa*. *PLoS ONE* **7**, e36442 (2012).
