# Supplementary material for: Transposon signatures of allopolyploid genome evolution
Source: Nat Commun. 2023 Jun 1;14:3180. doi: 10.1038/s41467-023-38560-z (PMC10235133; doi:10.1038/s41467-023-38560-z)
Supplement: Supplementary file 3 — Description of Additional Supplementary Files [file 41467_2023_38560_MOESM3_ESM.pdf]

## Description of Additional Supplementary Files

File Name: Supplementary Data 1
Description: ***Camelina* LTR families**
Zipped directory contains a .fasta file for the LTRs and inner sequence of retrotransposons identified by LTRHarvest. In addition, for each group of 13mers, there is a list of the retrotransposons that contain one or more 13-mers from that family. Additionally, the Camelina.LTR.subfam.tbl family files contains the independently called LTR subfamilies for each species, in addition to a list of LTRs included in that family. The Camelina.ltr.fam.tbl contains assignments of the LTRs to GyDB subfamilies.

File Name: Supplementary Data 2
Description: **Strawberry LTR families**
Zipped directory contains a .fasta file for the LTRs and inner sequence of retrotransposons identified by LTRHarvest. In addition, for each group of 13mers, there is a list of the retrotransposons that contain one or more 13-mers from that family. Additionally, the Strawberry.LTR.subfam.tbl family files contains the independently called LTR subfamilies for each species, in addition to a list of LTRs included in that family. The Strawberry.ltr.fam.tbl contains assignments of the LTRs to GyDB subfamilies.

File Name: Supplementary Data 3
Description: **Tobacco LTR families**
Zipped directory contains a .fasta file for the LTRs and inner sequence of retrotransposons identified by LTRHarvest. In addition, for each group of 13mers, there is a list of the retrotransposons that contain one or more 13-mers from that family. Additionally, the Tob.LTR.subfam.tbl family files contains the independently called LTR subfamilies for each species, in addition to a list of LTRs included in that family. The Tobacco.ltr.fam.tbl contains assignments of the LTRs to GyDB subfamilies.

File Name: Supplementary Data 4
Description: **Raw 13-mer tables**
For each species, we include a table that shows the 13- mer count per chromosome for 13-mers that occur >50 times in each genome. The columns are chromosomes (labeled in the header), and the rows are 13-mers.

File Name: Supplementary Data 5
Description: **Long-terminal repeat alignments in fasta format**
For each of tobacco, *Camelina*, and strawberry we include a subdirectory that includes the fastas of the multiple sequence alignment of LTR subfamilies. Additionally, each species has a *.familycount.tbl file that contains the # of LTRs assigned to each subgenome based on 13-mers in each family.

File Name: Supplementary Data 6
Description: ***B. napus* subgenome-specific 13-mers**
For *B. napus*, the spreadsheet shows the 13-mers in the first column, and the related subgenome in the second.

File Name: Supplementary Data 7
Description: **Cotton AD1 subgenome-specific 13-mers**
For *G. hirsutum*, the spreadsheet shows the 13-mers in the first column, and the related subgenome in the second.

File Name: Supplementary Data 8
Description: **Cotton AD2 subgenome-specific 13-mers**
For *G. barbadense*, the spreadsheet shows the 13- mers in the first column, and the related subgenome in the second.

File Name: Supplementary  Data 9
Description: **Carp subgenomespecific 13-mers**
For carp, the spreadsheet shows the 13-mers in the first column, and the related subgenome in the second.

File Name: Supplementary Data 10
Description: **Goldfish subgenomespecific 13-mers**
For goldfish, the spreadsheet shows the 13-mers in the first column, and the related subgenome in the second.

File Name: Supplementary Data 11
Description: ***Camelina*** **subgenomespecific 13-mers**
For *Camelina*, the spreadsheet shows the 13-mers in the first column, and the related subgenome in the second.

File Name: Supplementary Data 12
Description: **Strawberry subgenomespecific 13-mers**
For strawberry, the spreadsheet shows the 13-mers in the first column, and the related subgenome in the second.

File Name: Supplementary  Data 13
Description: **Tobacco subgenomespecific 13-mers**
For tobacco, the spreadsheet shows the 13-mers in the first column, and the related subgenome in the second.

File Name: Supplementary Data 14
Description: ***Arabidopsis*** **suecica subgenome-specific 13-mers**
For *A. suecica*, the spreadsheet shows the 13-mers in the first column, and the related subgenome in the second.
