# Supplementary material for: Transposon signatures of allopolyploid genome evolution
Source: Nat Commun. 2023 Jun 1;14:3180. doi: 10.1038/s41467-023-38560-z (PMC10235133; doi:10.1038/s41467-023-38560-z)
Supplement: Supplementary file 18 — Reporting Summary [file 41467_2023_38560_MOESM18_ESM.pdf]

nature portfolio

Corresponding author(s): Adam Session

Last updated by author(s): 2023-04-27

Reporting Summary

Nature Portfolio wishes to improve the reproducibility of the work that we publish. This form provides structure for consistency and transparency in reporting. For further information on Nature Portfolio policies, see our Editorial Policies and the Editorial Policy Checklist.

Please do not complete any field with "not applicable" or n/a. Refer to the help text for what text to use if an item is not relevant to your study.
For final submission: please carefully check your responses for accuracy; you will not be able to make changes later.

Statistics

For all statistical analyses, confirm that the following items are present in the figure legend, table legend, main text, or Methods section.

n/a	Confirmed	
☐	☑	The exact sample size (n) for each experimental group/condition, given as a discrete number and unit of measurement
☑	☐	A statement on whether measurements were taken from distinct samples or whether the same sample was measured repeatedly
☐	☑	The statistical test(s) used AND whether they are one- or two-sided<br>*Only common tests should be described solely by name; describe more complex techniques in the Methods section.*
☑	☐	A description of all covariates tested
☐	☑	A description of any assumptions or corrections, such as tests of normality and adjustment for multiple comparisons
☐	☑	A full description of the statistical parameters including central tendency (e.g. means) or other basic estimates (e.g. regression coefficient) AND variation (e.g. standard deviation) or associated estimates of uncertainty (e.g. confidence intervals)
☐	☑	For null hypothesis testing, the test statistic (e.g. F, t, r) with confidence intervals, effect sizes, degrees of freedom and P value noted<br>*Give P values as exact values whenever suitable.*
☑	☐	For Bayesian analysis, information on the choice of priors and Markov chain Monte Carlo settings
☐	☑	For hierarchical and complex designs, identification of the appropriate level for tests and full reporting of outcomes
☐	☑	Estimates of effect sizes (e.g. Cohen's d, Pearson's r), indicating how they were calculated

Our web collection on statistics for biologists contains articles on many of the points above.

Software and code

Policy information about availability of computer code

Data collection: Scripts at https://github.com/amsession/Kmer-based-Subgenome-Mapping/releases/tag/KmerSubgenome

Data analysis: Jellyfish v2.3, Rv4.05, bedtools v2.30, karyoploteR v1.26, BLAST+ 2.9.0

For manuscripts utilizing custom algorithms or software that are central to the research but not yet described in published literature, software must be made available to editors and reviewers. We strongly encourage code deposition in a community repository (e.g. GitHub). See the Nature Portfolio guidelines for submitting code & software for further information.

Data

Policy information about availability of data

All manuscripts must include a data availability statement. This statement should provide the following information, where applicable:

- Accession codes, unique identifiers, or web links for publicly available datasets
- A description of any restrictions on data availability
- For clinical datasets or third party data, please ensure that the statement adheres to our policy

Data supporting the findings of this work are available within the paper and its Supplementary Information files.
A reporting summary for this article is available as a Supplementary Information file.
Supplementary Data File 13 and the source data for individual figure panels are stored at the Dryad database under https://datadryad.org/stash/share/adtnfnCalUH1B

The genome data sets used in this study were obtained from publicly available databases.
The exact genome version and source for each species are in Supplemental Table 1.
Retrotransposon peptide consensi were obtained from the GyDB consensi (gydb.org).

Research involving human participants, their data, or biological material

Policy information about studies with human participants or human data. See also policy information about sex, gender (identity/presentation), and sexual orientation and race, ethnicity and racism.

Reporting on sex and gender	
Reporting on race, ethnicity, or other socially relevant groupings	
Population characteristics	
Recruitment	
Ethics oversight	

Note that full information on the approval of the study protocol must also be provided in the manuscript.

Field-specific reporting

Please select the one below that is the best fit for your research. If you are not sure, read the appropriate sections before making your selection.

☐ Life sciences ☐ Behavioural & social sciences ☐ Ecological, evolutionary & environmental sciences

For a reference copy of the document with all sections, see nature.com/documents/nr-reporting-summary-flat.pdf

Life sciences study design

All studies must disclose on these points even when the disclosure is negative.

Sample size	K-mer sample sizes are dependent on which k-mers are present in each assembly, as measured by Jellyfish. Genom
Data exclusions	No data excluded, Supplemental Data Files contain all datasets generated.
Replication	We study the full k-mer population for each species. Replication is not possible without independent genome asse
Randomization	We have no treatment groups and study the whole population of k-mers in each species, which does not allow ran
Blinding	There are no participants in our study, therefore blinding is not possible.

Behavioural & social sciences study design

All studies must disclose on these points even when the disclosure is negative.

Study description	No behavioral analysis done
Research sample	No behavioral analysis done
Sampling strategy	No behavioral analysis done
Data collection	No behavioral analysis done
Timing	No behavioral analysis done
Data exclusions	No behavioral analysis done
Non-participation	No behavioral analysis done
Randomization	No behavioral analysis done

Ecological, evolutionary & environmental sciences study design

All studies must disclose on these points even when the disclosure is negative.

Study description	We use differential k-mer density to identify subgenomes in polyploid species
Research sample	Polyploid genomes with known subgenomes were chosen to show the effectiveness of our method. Strawbe
Sampling strategy	Species were chosen based on the previous literature for the organism, no further subsampling was done.
Data collection	Genome data was downloaded from public databases.
Timing and spatial scale	Timing of the strawberry duplication was done by comparing our sequence change rates to published result
Data exclusions	No data was excluded
Reproducibility	Our results can be reproduced using the scripts provided, or using other methods that study k-mers.
Randomization	We have no treatment groups and study the whole population of k-mers in each species, which does not all
Blinding	There are no participants in our study, therefore blinding is not possible.

Did the study involve field work? ☐ Yes ☑ No

Field work, collection and transport

Field conditions	No field work done
Location	No field work done
Access & import/export	No field work done
Disturbance	No field work done

Reporting for specific materials, systems and methods

We require information from authors about some types of materials, experimental systems and methods used in many studies. Here, indicate whether each material, system or method listed is relevant to your study. If you are not sure if a list item applies to your research, read the appropriate section before selecting a response.

Materials & experimental systems

n/a	Involved in the study	
☑	☐	Antibodies
☑	☐	Eukaryotic cell lines
☑	☐	Palaeontology and archaeology
☑	☐	Animals and other organisms
☑	☐	Clinical data
☑	☐	Dual use research of concern
☑	☐	Plants

Methods

n/a	Involved in the study	
☑	☐	ChIP-seq
☑	☐	Flow cytometry
☑	☐	MRI-based neuroimaging

Antibodies

Antibodies used	No antibodies used
Validation	No antibodies used

Eukaryotic cell lines

Policy information about cell lines and Sex and Gender in Research

Cell line source(s)	No cell lines used
Authentication	No cell lines used
Mycoplasma contamination	No cell lines used
Commonly misidentified lines (See ICLAC register)	No cell lines used

Palaeontology and Archaeology

Specimen provenance	No paleontology or archeology
Specimen deposition	No paleontology or archeology
Dating methods	No paleontology or archeology

☐ Tick this box to confirm that the raw and calibrated dates are available in the paper or in Supplementary Information.

Ethics oversight	No paleontology or archeology

Note that full information on the approval of the study protocol must also be provided in the manuscript.

Animals and other research organisms

Policy information about studies involving animals; ARRIVE guidelines recommended for reporting animal research, and Sex and Gender in Research

Laboratory animals	No lab animals used
Wild animals	No wild animals used
Reporting on sex	No sex data was collected
Field-collected samples	No field samples collected
Ethics oversight	No animals or other organisms used

Note that full information on the approval of the study protocol must also be provided in the manuscript.

Clinical data

Policy information about clinical studies

All manuscripts should comply with the ICMJE guidelines for publication of clinical research and a completed CONSORT checklist must be included with all submissions.

Clinical trial registration	No clinical data used
Study protocol	No clinical data used
Data collection	No clinical data used
Outcomes	No clinical data used

Dual use research of concern

Policy information about dual use research of concern

Hazards

Could the accidental, deliberate or reckless misuse of agents or technologies generated in the work, or the application of information presented in the manuscript, pose a threat to:

No	Yes	
☑	☐	Public health
☑	☐	National security
☑	☐	Crops and/or livestock
☑	☐	Ecosystems
☑	☐	Any other significant area

Experiments of concern

Does the work involve any of these experiments of concern:

No	Yes	
☑	☐	Demonstrate how to render a vaccine ineffective
☑	☐	Confer resistance to therapeutically useful antibiotics or antiviral agents
☑	☐	Enhance the virulence of a pathogen or render a nonpathogen virulent
☑	☐	Increase transmissibility of a pathogen
☑	☐	Alter the host range of a pathogen
☑	☐	Enable evasion of diagnostic/detection modalities
☑	☐	Enable the weaponization of a biological agent or toxin
☑	☐	Any other potentially harmful combination of experiments and agents

Plants

Seed stocks	No plants used
Novel plant genotypes	No plants used
Authentication	No plants used

ChIP-seq

Data deposition

☐ Confirm that both raw and final processed data have been deposited in a public database such as GEO.

☐ Confirm that you have deposited or provided access to graph files (e.g. BED files) for the called peaks.

Data access links<br>*May remain private before publication.*	No ChIP-seq done
Files in database submission	No ChIP-seq done
Genome browser session<br>(e.g. UCSC)	No ChIP-seq done

Methodology

Replicates	No ChIP-seq done
Sequencing depth	No ChIP-seq done
Antibodies	No ChIP-seq done
Peak calling parameters	No ChIP-seq done
Data quality	No ChIP-seq done
Software	No ChIP-seq done

Flow Cytometry

Plots

Confirm that:

- [ ] The axis labels state the marker and fluorochrome used (e.g. CD4-FITC).
- [ ] The axis scales are clearly visible. Include numbers along axes only for bottom left plot of group (a 'group' is an analysis of identical markers).
- [ ] All plots are contour plots with outliers or pseudocolor plots.
- [ ] A numerical value for number of cells or percentage (with statistics) is provided.

Methodology

Sample preparation	No flow cytometry done
Instrument	No flow cytometry done
Software	No flow cytometry done
Cell population abundance	No flow cytometry done
Gating strategy	No flow cytometry done

- [ ] Tick this box to confirm that a figure exemplifying the gating strategy is provided in the Supplementary Information.

Magnetic resonance imaging

Experimental design

Design type	No MRI done
Design specifications	No MRI done
Behavioral performance measures	No MRI done

Imaging type(s)	No MRI done
Field strength	No MRI done
Sequence & imaging parameters	No MRI done
Area of acquisition	No MRI done

Diffusion MRI ☐ Used ☐ Not used

Preprocessing

Preprocessing software	No MRI done
Normalization	No MRI done
Normalization template	No MRI done
Noise and artifact removal	No MRI done
Volume censoring	No MRI done

Statistical modeling & inference

Model type and settings	No MRI done
Effect(s) tested	No MRI done

Specify type of analysis: ☐ Whole brain ☐ ROI-based ☐ Both

Statistic type for inference

(See Eklund et al. 2016)

No MRI done

Correction

No MRI done

Models & analysis

n/a	Involved in the study
☒	☐ Functional and/or effective connectivity
☒	☐ Graph analysis
☒	☐ Multivariate modeling or predictive analysis

Functional and/or effective connectivity

No MRI done

Graph analysis

No MRI done

Multivariate modeling and predictive analysis

No MRI done

This checklist template is licensed under a Creative Commons Attribution 4.0 International License, which permits use, sharing, adaptation, distribution and reproduction in any medium or format, as long as you give appropriate credit to the original author(s) and the source, provide a link to the Creative Commons license, and indicate if changes were made. The images or other third party material in this article are included in the article's Creative Commons license, unless indicated otherwise in a credit line to the material. If material is not included in the article's Creative Commons license and your intended use is not permitted by statutory regulation or exceeds the permitted use, you will need to obtain permission directly from the copyright holder. To view a copy of this license, visit http://creativecommons.org/licenses/by/4.0/
